# A new short version of the Facial expressions of emotion: Stimuli and tests (FEEST) including prototype and morphed emotional stimuli

**DOI:** 10.3389/fpsyg.2023.1198386

**Published:** 2023-10-24

**Authors:** Benedikt Kuhlmann, Jürgen Margraf

**Affiliations:** Department of Clinical Psychology and Psychotherapy, Mental Health Research and Treatment Center, Ruhr University Bochum, Bochum, Germany

**Keywords:** emotion recognition, prototype, morphed, short version, differences between emotions

## Abstract

The ability to recognize emotions from facial expressions plays an important role in social interaction. This study aimed to develop a short version of the FEEST as a brief instrument to measure emotion recognition ability by applying prototype and morphed emotional stimuli. Morphed emotional stimuli include mixed emotions. Overall, 68 prototypes and 32 morphed emotional expressions were presented to 138 participants for 1 s. A retest with 76 participants was conducted after 6 months. The results showed sufficient variance for the measurement of individual differences in emotion recognition ability. Accuracy varied between emotions and was highest for anger and happiness. Cronbach's α was, on average, 0.70 for prototypes and 0.67 for morphed stimuli. Test-retest reliability was 0.60 for prototypes and 0.62 for morphed stimuli. The new short version of the FEEST is a reliable test to measure emotion recognition.

## Introduction

The correct interpretation of the emotions of others is essential in human interaction, as facial expressions provide important insights into these emotions (Scherer and Ellgring, 2007; Todorov et al., 2011; Passarelli et al., 2018). Therefore, the ability to recognize emotions from facial expressions is an extremely crucial aspect of social cognition (Green et al., 2005; Liu et al., 2019). Some researchers suggest that the stability of emotion recognition ability is associated with personality traits like extraversion (Zuckermann et al., 1979) and creativity (Geher et al., 2017). Other studies demonstrated that the ability increases with age until young adulthood and decreases later in life (Charles and Campos, 2011; Kunzmann et al., 2018; Hayes et al., 2020). Additionally, the age of the face plays a role in emotion recognition (Fölster et al., 2014). Some evidence suggests that women may have slightly higher accuracy in emotion recognition compared to men (Connolly et al., 2019) and negative influences of psychological or neurological disorders on accuracy (Willis et al., 2010; Hoertnagl et al., 2011; Molinero et al., 2015). According to Surcinelli et al. (2022), emotions are detected more easily from facial expressions presented head-on than from faces presented in the profile. Emotion perception is often considered a lower-level cognitive process compared to the theory of mind, which includes conclusions on the mental state of others (Mitchell and Phillips, 2015).

Research on emotion recognition and its association with other abilities and disorders has a long history (Matsumoto et al., 2000). Over the decades, researchers have developed several behavioral instruments to measure emotional categorization ability (e.g., static, morphed, and dynamic facial stimuli, etc.), while in many psychopathological studies, non-validated tests were applied (de Paiva-Silva et al., 2016). However, the most frequently used stimuli to test emotion recognition are static facial photos by Ekman and Friesen called “Pictures of facial affect” (1976, 1978). Their database has served as a basis for new tasks comprising black-and-white photos of adult male and female faces expressing six universal emotions (anger, fear, sadness, happiness, disgust, and surprise).

The FEEST by Young et al. (2002) adopts these stimuli to test the emotion recognition ability in normal, psychiatric, and neurological populations. It includes the Ekman 60 Faces Test (Ekman and Friesen, 1976), which tests the recognition of the six basic emotions shown by 10 people (6 female and 4 male faces). The test includes practice trials; each stimulus is shown for 5 s. Additionally, it includes the emotion hexagon test using computer-manipulated mixed (morphed) emotional expressions (e.g., 80% anger, 20 % disgust). By morphing facial stimuli, the created faces are closer or more distant with regard to the prototypes. Morphed emotional stimuli were originally developed to examine categorical perceptions of facial expressions. Even though they are unnatural (Montagne et al., 2007), they allow us to measure subtler deficits in emotion recognition (de Paiva-Silva et al., 2016). The emotion hexagon test consists of five test blocks of 30 trials. Generally, accuracy in emotion recognition increases with the intensity of the emotion (Young et al., 2002). The third part of the FEEST is the emotional megamix, which includes all possible continua in 10% of the steps; thus, there are nine intensities in total.

The level of difficulty depends on how similar the morphed stimulus is with respect to the prototype stimulus. The hexagon test includes the six emotional continua, which are supposed to show the highest confusability rate (happiness; surprise, surprise; fear, fear; sadness, sadness; disgust, disgust; anger, anger; happiness) (Calder et al., 1996).

The broad application of the FEEST over 30 years reflects its reliability (Allen-Walker and Beaton, 2014). However, the fact that participants have to respond to many stimuli over a long period of time could reduce attention and impede the recruitment of participants, especially if associations with other variables are assessed. Therefore, many studies apply only a selected set of stimuli from Ekman and Friesen (1976). The Facial Emotion Identification Test (Kerr and Neale, 1993), for example, includes a selected number of stimuli by Ekman and Friesen (1976) and shows acceptable reliability (Combs and Penn, 2004). Only two studies on short versions of the FEEST have applied morphed stimuli. Gagliardi et al. (2003) applied four intensities of five emotions to assess facial expression recognition in Williams Syndrome. Montagne et al. (2007) applied a test using dynamically morphed emotional stimuli with nine different intensities (from 10% to 100%) and four stimuli per intensity to measure subtle deficits in emotion recognition. In addition to the finding of higher accuracy with higher emotional intensity, accuracy was highest for happiness and lowest for fear.

To the best of our knowledge, to date, there is no reliable short test of the FEEST applying a balanced number of prototypes and morphed stimuli. The use of more morphed emotional stimuli enables a better measurement of subtle deficits in emotion recognition.

The goal of the current study was to develop a quick, consistent, and reliable version of the FEEST as the first test with a balanced selection of prototype stimuli and morphed emotional stimuli. The resulting test was supposed to be a useful measure to detect individual differences in emotion recognition and deficits in emotion recognition in the general population. The reduction in time needed to complete the task will facilitate further research on associations between facial emotion recognition and other variables. As previous research shows differences in accuracy between emotions and depending on emotional intensity, a detailed analysis of accuracy is necessary to verify if this short version can measure these differences accurately.

A lower accuracy for morphed emotions compared to prototype emotions was hypothesized. Additionally, we expect higher accuracy for happiness compared to other emotions. Moreover, acceptably high internal consistency was expected.

## Method

### Emotional face task

Overall, 68 stimuli were selected from FEEST (Young et al., 2002) and presented in a randomized order. The task consisted of two different blocks with six practice trials before each block. Block 1 included a selection of 36 prototype stimuli from the Ekman 60 faces test, comprising six stimuli (three male and three female faces) for each of six basic emotions (anger, fear, happiness, sadness, surprise, and disgust). The stimuli were chosen from the Ekman 60-face set, which includes six female and four male faces for every prototype emotion. For every emotion, the stimuli with the numbers 1, 2, and 5 (female faces) and numbers 7, 9, and 10 (male faces) were chosen systematically. Further, 32 morphed emotional stimuli were selected from the Emotion Hexagon Test, including the stimuli of graded mixed emotions, for example, 80% anger and 20 % disgust. To reduce the time burden for participants and ceiling effects, only the four continua that show the highest confusability rates according to Calder et al. (1996) (anger-disgust, disgust-sadness, fear-sadness, and fear-surprise) were selected from the database. To reduce the time required, we included only four intensities per continuum: 80% vs. 20%, 60% vs. 40%, 40% vs. 60%, and 20% vs. 80%. Each emotion was shown by both a female (acronym “MO”) and a male (acronym “JJ”) face at each of the named intensities.

### Procedure

The study was conducted entirely online. Participants were sent a link and a password with which they could enter the task. Participants were informed before through an information sheet that they have to perform a task on emotion recognition for a duration of 10–15 min, and they should ensure they can solve the task in a quiet environment without interruptions. They were asked to wear their spectacle lenses during the task if they regularly wear them.

The procedure for the task:

Before the trial, a fixation cross was shown for 1 s. In Nook et al. (2015), the average reaction time for giving a response to an emotional stimulus was under 1 s. To shorten the time required for the task, we reduced the presentation time of each stimulus to 1 s, unlike in other applications of the FEEST. After the cue, a black screen was shown for 1 s. Afterward, participants had to give their responses within 7 s by clicking on the relevant term.

Contrary to the FEEST, the emotion names were shown after the stimuli. Initially, participants were instructed to choose the emotion that would best match the shown facial expression for each stimulus and to give their answers as fast as possible. The terms for the six basic emotions were shown in a hexagonal shape and in the same order on the screen. As the cursor was automatically located at the center of the screen at the beginning of each trial, the hexagonal shape of the list of emotions ensured that the distance between the cursor and each term was equal and that the location was familiar from the practice trials. After 7 s, the subsequent trial started automatically. The order of the two blocks was randomized.

After 4–6 months, participants were contacted again via email and asked to perform the same task again on their PC. The link and password were sent to participants in another email. The procedure for the face task at the second test was the same as the first.

### Participants

The 147 participants who provided informed consent were students from Ruhr-Universität Bochum, Germany, and were recruited via advertisements at the university. Most of the participants were psychology students who gained course credit by participating.

Apart from two Asian participants, all other participants were of Caucasian origin. Data from four participants were excluded because their ages were more than two standard deviations higher than the average age of 23.55 years. There were missing results in the emotional face task for five participants. Of the remaining 138 participants included in the analysis, the mean age was 22.9 years (SD = 4.81), with an age range of 18–38 years; 104 participants were women, and 34 were men. Of them, 76 took part in the second testing after 4–6 months. In addition, 73 of these (55 women and 18 men) completed the full study, while data from the emotional face task was missing for two of them. The study was conducted entirely online; participants were sent links with a personal password to access the face-to-face tasks and questionnaires. The participants completed the task on their PCs.

## Results

### Accuracy

**Figure 1:**
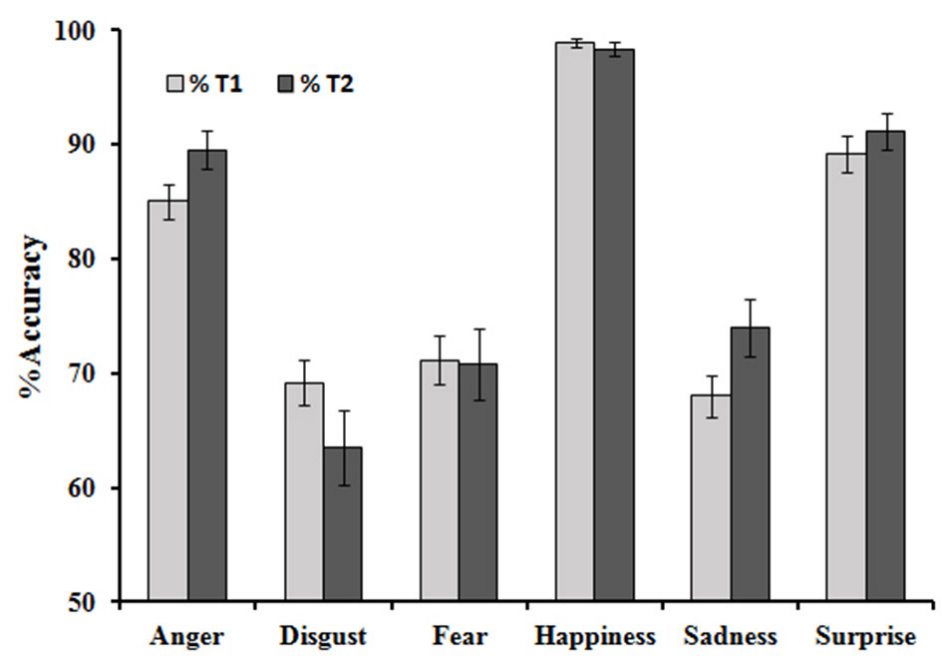
Accuracy for prototype emotions at both measuring points. Vertical bars represent the standard error of the means.

There was an average of 75.04% (SD = 9.48) correct answers. At the first measuring point, participants showed an average of 80.51% (SD = 9.51) correct responses for the prototype emotions, with an average of 0.95 (SD = 2.78) missing values. There were 68.28% (SD = 12.83) correct responses for the morphed stimuli and 1.06=% (SD = 3.12) missing values. As participants were instructed to choose the emotion that fit the most, anger was the correct answer for a stimulus, with 60% anger and 40% disgust. A paired-sample *t*-test was conducted to compare the percentage of correct answers in both blocks and showed a significant difference between both blocks [*t*_(137)_ = 13.38, *p* < 0.001]. There were no significant correlations of demographic variables such as age, *r* = −0.11, *n* = 138; *p* = 0.17, and education level, *r*_(136)_ = 0.04, *n* = 138, *p* = 0.63, with accuracy. An independent *t*-test was conducted to compare the number of correct answers between male participants (M = 49.62, SD = 7.91) and female participants (M = 51.55, SD = 5.87) and showed no significant differences, *t*_(136)_ = 1.47, *p* > 0.05. Table 1 shows the observed and possible range for all emotions and differentiates between positive vs. negative and prototype vs. morphed emotions. Figure 1 shows the mean accuracy for all prototype emotions at both measuring points.

**Table 1:** Accuracy in emotional face task showing possible accuracy range, accuracy for first test (T1), and second test after 6 months (T2), including mean and standard deviation (SD).

**Variable**	**Possible range**	**T1 observed range**	**T1 M (SD)**	**T2 observed range**	**T2 M (SD)**
Accuracy for all facial expressions	0–68	26–62	51.03 (6.45)	30–63	51.63 (6.55)
Total “prototype” (*N* = 139)	0–36	15–36	29.12 (3.40)	14–35	29.08 (3.64)
Total “morphed” (*N* = 139)	0–32	1–29	21.91 (3.97)	12–29	22.55 (3.74)
Accuracy for all **positive** emotions	0–16	5–16	14.36 (1.69)	8–16	14.22 (1.77)
Accuracy for all **negative** emotions	0–52	10–48	36.22 (6.60)	18–48	37.41 (6.28)
Accuracy for all **anger** expressions	0–10	0–10	8.30 (1.71)	5–10	8.74 (1.24)
Accuracy for all prototype anger expressions	0–6	0–6	5.10 (1.05)	2–6	5.37 (0.84)
Accuracy for all morphed anger expressions	0–4	0–4	3.20 (0.95)	1–4	3.37 (0.81)
Accuracy for all **disgust** expressions	0–14	0–14	9.51 (3.09)	1–14	9.38 (3.12)
Accuracy for all prototype disgust expressions	0–6	0–6	4.15 (1.41)	0–6	3.81 (1.64)
Accuracy for all morphed disgust expressions	0–8	0–8	5.35 (2.20)	0–8	5.59 (1.94)
Accuracy for all **fear** expressions	0–14	0–14	9.62 (2.86)	0–14	9.73 (2.94)
Accuracy for all prototype fear expressions	0–6	0–6	4.27 (1.49)	0–6	4.25 (1.59)
Accuracy for all morphed fear expressions	0–8	0–8	5.35 (1.92)	0–8	5.48 (1.89)
Accuracy for all **happy** expressions	0–6	4–6	5.93 (0.28)	5–6	5.90 (0.296)
Accuracy for all **sadness** expressions	0–14	0–14	8.80 (2.71)	2–14	9.56 (2.67)
Accuracy for all prototype sadness expressions	0–6	0–6	4.08 (1.31)	1–6	4.44 (1.29)
Accuracy for all morphed sadness expressions	0–8	0–8	4.72 (1.84)	0–8	5.12 (1.77)
Accuracy for all **surprise** expressions	0–10	0–10	8.43 (1.58)	3–10	8.43 (1.58)
Accuracy for all prototype surprise expressions	0–6	0–6	5.35 (1.09)	3–6	5.47 (0.80)
Accuracy for all morphed surprise expressions	0–4	0–4	3.04 (0.95)	0–4	2.85 (1.16)

A GLM for the prototype stimuli with repeated measures and accuracy as dependent variables showed a main effect of emotion, *F*_(4.05/554.39)_ = 67.50, *p* < 0.001. $\eta_{p}^{2}$ = 0.330. *Post-hoc* sample *t*-tests were calculated with a Bonferroni adjusted alpha of 0.003 (*p* = 0.05/15) to compare accuracy for each emotion with 15 pairs in total. Accuracy for anger was significantly higher than for disgust [*t*_(137)_ = 6.69, *p* < 0.001, *d* = 0.57], fear [*t*_(137)_ = 6.03, *p* < 0.001, *d* = 0.51], and sadness [*t*_(137)_ = 7.99, *p* < 0.001, *d* = 0.68].

Accuracy for happiness was higher than that for fear [*t*_(137)_ = 13.35, *p* < 0.001, *d* = 1.14], sadness [*t*_(137)_ = 16.93, *p* < 0.001], surprise [*t*_(137)_ = 6.60, *p* < 0.001], disgust [*t*_(137)_ = 14.82, *p* < 0.001, *d* = 1.26], and anger [*t*_(137)_ = 10.16, *p* < 0.001, *d* = 0.87]. Accuracy for surprise was higher than that for fear [*t*_(137)_ = 7.03, *p* < 0.001, *d* = 0.59], sadness [*t*_(137)_ = 9.95, *p* < 0.001, *d* = 0.85], and disgust [*t*_(137)_ = 8.77, *p* < 0.001, *d* = 0.75].

Accuracy did not differ significantly between anger and surprise [*t*_(138)_ = −2.09; *p* > 0.003], disgust and fear [*t*_(138)_ = −0.699, *p* > 0.003], disgust and sadness [*t*_(138)_ = 0.463, *p* > 0.003], fear and sadness [*t*_(137)_ = 1.297, *p* > 0.003].

Table 2 shows the mean accuracy for the four different continua. A repeated measure GLM was conducted with each continuum as the independent variable and accuracy as the dependent variable and showed a significant difference between accuracy at the different continua, *F*_(3, 411)_ = 5.26: *p* = 0.001, $\eta_{p}^{2}$ = 0.037. Post-hoc paired sample *t*-tests were calculated to compare the different continua with a Bonferroni adjusted alpha of 0.008 (*p* = 0.05/6) for six comparisons and showed that accuracy at Continuum 1 (anger-disgust) was significantly higher than that for Continuum 2 (disgust-sadness), *t*_(137)_ = 4.04, *p* < 0.008, *d* = 0.34, and Continuum 3 (fear-sadness), *t*_(137)_ = 2.93, *p* = 0.004, d = 0.25. The differences between Continuum 1 (anger-disgust) and Continuum 4, *t*_(137)_ = 2.47, *p* > 0.008, Continuum 2 (disgust-sadness) and Continuum 3 (fear-sadness), *t*_(137)_ = −0.60, *p* > 0.008, Continuum 2 (disgust-sadness) and Continuum 4, *t(137)* = −0.12, *p* > 0.008, and Continuum 3 (fear-sadness) and Continuum 4 (fear-surprise), *t*_(137)_ = −0.61, *p* > 0.008, were not significant.

**Table 2:** Accuracy at emotion recognition for the different continua showing mean (M) of correct answers and standard deviations (SD).

**Continuum**	**M Correct answer (SD)**
Anger- disgust (continuum 1)	5.78 (1.55)
Disgust- sadness (continuum 2)	5.23 (1.56)
Fear-sadness (continuum 3)	5.32 (1.47)
Fear-surprise (continuum 4)	5.41 (1.35)

The possible range was 0–8 correct answers for every continuum.

### Factor analysis

To explore the factorial structure of this short version of the FEEST, the scales were subjected to an exploratory factor analysis. The Kaiser-Meyer-Olkin measure showed sampling adequacy for the analysis, KMO = 0.7. Bartlett's test of sphericity showed that the data are adequate for factor analysis, *t*_(45)_ = 246.22, *p* < 0.001. The analysis found a three-factor solution, including all variables with eigenvalues >1. Table 3 shows the factor loadings. An analysis of the eigenvalues, however, shows a dramatic drop in these values after the first factor. Specifically, the eigenvalue for factor 1 stands at 3.03, while for factor 2, it is 1.24, and for factor 3, it is 1.14. Given that factors 2 and 3 contribute minimal additional information and pose challenges in interpretation, a one-factor solution appears more appropriate for this analysis.

**Table 3:** Exploratory factor analysis.

	**Components**		
	**1**	**2**	**3**
AngerB1	**0.538**	0.022	−0.63
DisgustB1	0.41	**0.482**	**0.455**
FearB1	**0.522**	−0.445	−0.018
HappinessB1	**0.547**	0.332	−0.512
SadnessB1	**0.559**	0	−0.008
SurpriseB1	**0.458**	**0.464**	−0.074
Anger-disgust	**0.715**	0.188	0.093
Disgust-sadness	**0.683**	0.084	0.4
Fear-sadness	**0.553**	−0.56	−0.001
Fear-surprise	**0.444**	−0.198	0.319

Factor loadings for three components of the model.

Factor loadings larger than 0.40 are in bold.

### Internal consistency

Based on the findings of this factor analysis and the fact that the items are dichotomous, the Kuder-Richardson formula 20 was used to calculate the internal consistency of the whole test. The KR 20 value for the whole test was 0.800. The KR 20 value for the prototype stimuli was 0.703 and for the morphed stimuli 0.67.

### Retest

At the second measuring point, the participants showed an average of 80.78% (SD = 10.11) of correct answers for the prototype stimuli and 70.46% (SD = 11.70) for the morphed stimuli. The total percentage of correct answers at the second measuring point was 75.93% (SD = 9.64). See Table 1 for observed ranges and mean accuracy for retest data T2.

The test-retest reliability for the number of correct answers for the whole test was *r* = 0.712, *p* < 0.001, for prototype stimuli, *r* = 0.600, *p* < 0.001, and for morphed stimuli, *r* = 0.623, *p* < 0.001. See Table 4 for the test-retest reliability for all emotions.

**Table 4:** Test-retest reliabilities for prototype and morphed emotional stimuli.

**Emotion**	**Test-retest-reliability**	
	**Prototype**	**Morphed**
Anger	0.337^**^	0.024
Disgust	0.373^**^	0.568^**^
Fear	0.541^**^	0.534^**^
Happiness	0.330^**^	
Sadness	0.457^**^	0.448^**^
Surprise	0.296^*^	0.543^**^
total	0.600^**^	0.623^**^

^*^p < 0.05.

^**^p < 0.005.

## Discussion

The current study aimed to check the reliability of a short version of the FEEST, including both prototype and morphed stimuli. On the whole, the results showed sufficient variance to measure individual differences in emotion recognition. There are differences in accuracy depending on emotion, with medium to high effect sizes. The hypothesis that morphed stimuli's accuracy would be lower than prototype stimuli was confirmed: morphed emotions are more difficult to distinguish from each other.

The results for prototype stimuli by Ekman and Friesen (1976) are similar to this study; both studies show over 80% accuracy. In Ekman and Friesen (1976), accuracy for some emotions was higher than for others. Happiness showed a ceiling effect, and accuracy was lowest for fear. The ceiling effect for the emotion of happiness could be confirmed in the current sample. The current sample's accuracy for fear, disgust, surprise, and sadness was lower than that of Ekman and Friesen (1976). However, accuracy for anger was higher in the current sample.

The differences depending on emotion are also supported by other findings. A recent study by Molinero et al. (2015) used the Ekman 60 Face test in Spanish adolescents and also reported the highest accuracy for happiness and the lowest accuracy for anger and fear. Regarding the morphed stimuli, accuracy for the continuum anger-disgust, which showed the highest confusability rates in Calder et al. (1996), was surprisingly higher than that for all other continua in this study. This finding corresponds to higher accuracy for anger in the prototype stimuli in the current study. Garcia and Tully (2020) reported higher accuracy in recognizing anger than fear in 7- to 10-year-old children. Montagne et al. (2007) reported the highest accuracy for happiness and anger in morphed stimuli. Garcia and Tully (2020) explain these differences in terms of the functionality of emotions. Anger expresses a personal threat to the viewer and thus might be more salient than other emotions and can still be detected in morphed conditions.

Overall, differences in accuracy between emotions point to the multidimensionality of emotion recognition ability, which is supported by other findings (Calder et al., 1996; Sprengelmeyer et al., 1997; Passarelli et al., 2018). Happiness is easier to recognize than other emotions. Contrary to other studies (e.g., Molinero et al., 2015), the results showed only a tendency for higher accuracy among female participants compared to male participants. However, this can be due to the low number of male participants.

Despite the multidimensionality, only one factor could be detected in the exploratory factor analysis, even though the majority of the variance could not be explained by this factor.

The level of internal consistency of the whole test is high enough to be used for future research. Internal consistency was lower for the morphed stimuli.

This might be due to the reduced number of morphed stimuli in this study compared to the FEEST. The low Cronbach's α values, if differentiated between continua, might be due to the small number of items in each continuum.

The test-retest reliability of the overall face task was acceptable but moderate for anger, disgust, happiness, and surprise. Cecilione et al. (2017) examined the test-retest reliability of the facial expression labeling task (FELT) in children, varying the expressivity of the emotional stimuli. The interval between the two tests was 2–5 weeks. They reported slightly lower test-retest reliability (between 0.39 and 0.54) for highly expressive stimuli compared to the current study. Reliability increased when emotions were easier to recognize. In the current study, however, the test-retest reliability was comparable for the prototype and morphed stimuli. Lo and Siu (2018) assessed the test-retest reliability for the Face Emotion Identification Test and found reliability higher than 0.75 with a 1-week interval. The lower test-retest reliability in the current sample can be explained by the longer interval between the two measuring points. The ability to recognize emotions might alter over time.

### Limitations

The use of static stimuli is a limitation of this study, as social stimuli in real-life situations are dynamic. Additionally, the fact that the stimuli are already over 40 years old has to be mentioned. However, its reliability has been proven by its frequent use in past and current research. The moderate internal consistency of the morphed emotional stimuli due to the reduced number of emotional stimuli is another limitation of this study. Since there is some evidence of better performance by women in emotional face tasks, the higher number of female participants is another limitation of the study, even though the differences are small and limited to facial disgust (Connolly et al., 2019). The fact that all participants completed the task on their PC is another limitation, as uncontrolled variables could have influenced the results.

Overall, the current results show that this short version of the FEEST is a reliable measure of the ability to recognize emotions from facial expressions for both prototype and morphed stimuli.

## Data availability statement

The raw data supporting the conclusions of this article will be made available by the authors, without undue reservation.

## Ethics statement

The studies involving humans were approved by Ethikkommission der Fakultät für Psychologie der Ruhr Universität Bochum. The studies were conducted in accordance with the local legislation and institutional requirements. The participants provided their written informed consent to participate in this study.

## Author contributions

All authors listed have made a substantial, direct, and intellectual contribution to the work and approved it for publication.
